# Supplementary material for: The impact of city-county merger on firm-level total factor productivity—Evidence from China
Source: PLoS One. 2024 Sep 16;19(9):e0304129. doi: 10.1371/journal.pone.0304129 (PMC11404814; doi:10.1371/journal.pone.0304129)
Supplement: Attachment — Submitted filename: Response to review #1.docx [file pone.0304129.s001.docx]

**The Impact of City-county Merger on Firm-level Total Factor Productivity**

**Response to review #1**

We sincerely appreciate your engagement and time in reviewing our manuscript as well as your suggestions on improving it. Below there is a detailed account of how we have revised the manuscript based on each of your suggestions.

**Comment 1:** *In the robust test (in section 5.2.4), this paper exclusively employs the CSDID method to tackle the heterogeneous treatment effects of the Two-Way Fixed Effects (TWFE) estimator in the baseline under static conditions. However, it is equally crucial to derive the firm-year average treatment effect under dynamic conditions, as shown in Figure 5. Moreover, there have been several proposed "heterogeneous-robust" estimators that warrant further verification.*

**Response to comment 1:** Thank you for your valuable suggestions. In recent years, many scholars have observed the issue of heterogeneous treatment effects in Staggered DID, indicating that the traditional TWFE estimates may be biased even when the parallel trend assumption is satisfied[1-4]. To address the problem of varying treatment effects in Staggered DID, scholars have developed a range of "heterogeneous-robust estimators". In this paper, we address this issue by adopting the CSDID method developed by Callaway & Sant'Anna[3] and selecting the Never Treated Group and the Not-Yet-Treated Group as control groups, respectively. To further demonstrate the robustness of the conclusions in this paper, we incorporate the DIDM estimator proposed by De Chaisemartin & D'Haultfoeuille [1] as well as the Stacked Regression Estimator proposed by Cengiz at al. [5] to rectify the Staggered DID and exhibit dynamic treatment effects. The test results are depicted in the figure as follows, indicating no significant difference between the coefficients of the treatment and control groups in periods prior to the policy, further bolstering the robustness of the baseline regression results. The specific modifications are outlined in the new section 5.2.5: Heterogeneous Treatment Effects, which has been added to the revised manuscript (page 19 of the revised manuscript).

Figure 1. Two types of DID estimators

**Comment 2:** *The sample numbers for some columns of regression were inconsistent due to missing value of control variables or other variables, such as in table 2, columns (1)-(4); table 3, columns (1)-(4); table 4, columns (1)-(4); table 6, column (6); table 8, columns (1)-(5); table 9, columns (1)-(5).*

**Response to comment 2:** Indeed, we clarify the observed changes in sample sizes. As highlighted by the reviewer, the absence of values in some control variables leads to variations in sample sizes between regressions with and without control variables, as depicted in Tables 2, 3, and 4. To address this concern, we standardize the sample size to 411,575 (derived from the baseline regression with all control variables and fixed effects included). As a result, the sample numbers in Tables 2, 3, and 4 have remained consistent. The differences in sample numbers in Table 6 arises from the automatic removal of a small number of samples in the regression when controlling for the cross-multiplier terms of region and industry. However, when we use the sample of 411,575 to run the regressions, the results in (1) - (7) of Table 6 are almost identical. Tables 8 and 9 examine the impact of TCD reforms on the government's fiscal environment and firms' internal environment. On one hand, each explanatory variable involved has some missing data. On the other hand, in the case of large sample regressions, convergence necessitates different numbers of iterations, resulting in a loss of sample size. Therefore, the inconsistency in sample size in Tables 8 and 9 is inevitable.

**Comment 3:** *In table 6, according to the author's explanation, policy 1 (strengthen counties by expanding their powers) and policy 2 (province-managing county) both enhanced the administrative power of the county. However, the coefficients for policy 1 unexpectedly appear negative in columns (3) and (4), whereas the coefficients for policy 2 are positive in both columns (3) and (4).*

**Response to comment 3:** Thank you for your valuable suggestions. Both policy1 (Province-managing County (PMC)) and policy2 (strengthen counties by expanding their powers) delegate the governmental authority to counties, albeit through different mechanisms. Policy2 entails the transfer of certain economic and social management powers from prefectural cities to counties, including project and investment approval. By contrast, policy1 centers on the allocations of fiscal revenues and expenditures to establish a direct connection between the province and the county, thereby increasing the fiscal authority of county governments and eliminating the role of prefecture city administration as an intermediary between the province and the county. More specifically, policy1 can be seen as a type of governmental management restructuring that shifts from a "province-city-county" management system to a "province-county" management system. This restructuring amplifies the fiscal revenue of counties. However, given that the number of counties far exceeds the number of prefecture-level cities, PMC reform greatly increases the number of administrative units that the province needs to manage horizontally. This escalation in the level of management span diminishes the supervisory and coordinating capabilities of the provincial government, leading to a rise in illegal activities such as land corruption and misappropriation of public funds[6, 7]. Therefore, although PMC expands the financial power of counties and improves their financial income, it lowers the efficiency of county governments, consequently lowering firm-level TFP.

Thanks again to you for your valuable comments. We have elucidated the negative regression coefficients of the PMC reforms, with the findings outlined in section 5.2.6 of the revised manuscript (page 21 of the revised manuscript).

**Comment 4:** *The mechanism analysis through which the TCD reform can impact firm-level TFP is not clear and direct. According to the authors’ explanation, the TCD reform has had an impact on the government’s fiscal situation (checked in table 8), leading to changes in (institutional and non-institutional) transaction efficiencies (explained in section 2). Moreover, the changes in transaction efficiency from city-county mergers can influence firm performance (checked in table 9), leading to variations in productivity. However, the inframarginal model developed by Yang & Shi (1992) only explores how the changes in transaction efficiency from county-city mergers affect firm-level TFP. I wonder what role the model plays in the mechanism of firms’ internal environment, and there is a lack of a detailed explanation of how adjustments in the external environment might trigger changes in the internal circumstances of firms.*

**Response to comment 4:** Thank you for your valuable suggestions. The theoretical section of this paper proposes that centralized TCD reforms leads to a decline in institutional transaction efficiency, thereby causing a decrease in firm-level TFP. In effect, changes in government fiscal revenue and expenditure demonstrate the government's ability to regulate transaction efficiency. Therefore, the first part of the mechanism test examines the impact of the TCD reforms on the government's fiscal situation. As shown in Table 8, after TCD reforms, the government's fiscal situation has worsened in both revenue and expenditure, indicating a decrease in the government's ability to improve transaction efficiency and subsequently reduce firm-level TFP. Moreover, as a result of the TCD reforms, county-level governments have seen decreases in their authority over economic, social, and financial management, as well as changes in their policy priorities. This has led to decreased efficiency in institutional transactions and a reduction in the financial support (such as tax incentives and fiscal subsidies) that county-level governments can offer to businesses. The post-reform adjustments indicate that firms would face a shift in the external policy environment, highlighting the external impact of the TCD reforms on firm operations.

In effect, the inframarginal model developed by Yang and Shi [8] fails to address the relationship between changes in external transaction efficiency and the internal environment of firms. However, such changes in the external environment directly affect firms' operational performance [9]. Additionally, the investments made by firms to improve TFP are uncertain and risky [10], creating higher demands on firms’ operational and cash flow conditions. In this context, section 6.2 explores an alternate pathway through which TCD reforms impact firm-level TFP: TCD reforms deteriorate firms' internal environments, subsequently causing a decline in firm-level TFP.

Thanks again for your insightful remarks on this section. In the updated manuscript, we have made adjustments to the description of shifts in the firms' internal environment in section 6.2 (page 26 of the revised manuscript). The detailed modifications are as follows.

Changes in the external environment can directly impact firm performance[9]. Williamson [11] emphasizes the close relationship between government regulation and firms’ operations. Some researchers have discovered that government fiscal policies can significantly affect firms’ operational activities[12-14]. Total factor productivity (TFP) is a crucial indicator of firm performance and is naturally influenced by changes in the external environment. Aghion et al. [15] find that the tax burden on firms can affect the increase in TFP. Moreover, firms' investment in improving TFP is unpredictable and risky [10], creating higher demands on firms’ operational and cash flow conditions. Therefore, the operational capacity and financing constraint of firms also exert significant impact on the firm-level TFP [16-17].

As previously mentioned, the TCD reform has impacted the government's fiscal situation, resulting in alterations in transaction efficiencies. Furthermore, as authority shifts, there will be a reduction in local government revenue and therefore less incentive to promote economic development. For instance, the local government may decrease tax incentives and other supportive policies for businesses, implying that the TCD reforms have worsened the external environment for firm operations. Consequently, these changes in the external environment could lead to adjustments in the internal circumstances of firms, including tax burden, return on assets, and financing constraints, ultimately affecting firm-level TFP.

**Comment 5:** *Regarding the mechanism of negative effects, another possible explanation is that the city-county mergers have strengthened the administrative jurisdiction of the municipal government over the counties, reduced administrative barriers, promoted the smoother flow of various resource elements such as capital and labor, and affected the entry and exit behavior of enterprises. In other words, it has led to the entry of some less efficient enterprises in a short period of time, or the exit of more efficient enterprises.*

**Response to comment 5:** We are very grateful for your comments on the mechanism of this paper, as it offers a clear roadmap for enhancing the paper further. In response to the reviewers' suggestions, we have incorporated additional content in section 6 (page 27 of the revised manuscript). The specifics are outlined below.

City-county mergers can help break down administrative barriers between regions, strengthen connections between different areas, and facilitate the flows of capital, labor, and other factors. However, because new municipal areas are often located on the outskirts of cities, prefecture governments may not focus as much on new municipal areas as they do on old ones. Additionally, the older municipal areas are often economically stronger than the new ones. As a result, after the reforms, the old municipal areas may siphon more production factors, exacerbating the "core-periphery" effect [18]. This could lead to high-productivity firms migrating to the older areas, while the new municipal areas only attract low-productivity firms. Finally, the decrease in firm-level TFP after the TCD reforms is probably owing to the entry-exit behavior of firms.

To test this mechanism, this paper separately builds the cross-multipliers of policy and firm-entry (*policy*entry*), and firm-exit (*policy***exit*), respectively.^[1]^ The results are shown in the table 10. The coefficient of *policy*entry* in column (1) suggests that firm entry exacerbates policy effects, indicating that the entry of inefficient firms leads to a reduction in firm-level TFP in the reformed county-level cities. The coefficient of *policy***exit* in column (2) is statistically significant at 1% level, indicating that firms' exit behavior also amplifies the negative impact of TCD reforms on firm-level TFP. Additionally, we test the impact of the TCD reforms on non-entry and non-exit firms. Columns (3) and (4) show that the coefficients of policy remain significantly negative after excluding the effects of entry and exit firms, respectively, suggesting that the TCD reforms also have a negative effect on TFP for continuing firms.

Table 1 the impact of the entry-exit behavior of firms

|  | (1) | (2) | (3) | (4) |
| --- | --- | --- | --- | --- |
|  | TFP | TFP | TFP | TFP |
| policy | -0.042*** | -0.024* | -0.042*** | -0.024* |
|  | (-3.53) | (-1.71) | (-3.51) | (-1.71) |
| policy*entry | -0.862*** |  |  |  |
|  | (-2.69) |  |  |  |
| policy*exit |  | -0.083*** |  |  |
|  |  | (-3.48) |  |  |
| Constant | 5.514*** | 5.498*** | 5.501*** | 5.499*** |
|  | (525.89) | (589.22) | (593.32) | (586.87) |
| Control variables | YES | YES | YES | YES |
| Firm FE | YES | YES | YES | YES |
| Year FE | YES | YES | YES | YES |
| Industry FE | YES | YES | YES | YES |
| Observations | 411,575 | 411,575 | 405,218 | 407,734 |
| R-squared | 0.793 | 0.793 | 0.793 | 0.793 |

**Comment 6:** *Typos. In section 6.2, please note the absence of parentheses in several instances. Change “Column 1” to “Column (1)” for consistency, as well as “Column 2”, ..., and “Column 5”, etc."*

**Response to comment 6:** Thank you for your valuable suggestions. We have made modifications to the formatting issues throughout the text in the revised manuscript.

**reference**

1.	De Chaisemartin C, D’Haultfœuille X. Two-way fixed effects and differences-in-differences estimators with several treatments. Journal of Econometrics. 2023;236(2):105480.

2.	Goodman-Bacon A. Difference-in-differences with variation in treatment timing. Journal of Econometrics. 2021;225(2):254-77.

3.	Callaway B, Sant’Anna PHC. Difference-in-Differences with multiple time periods. Journal of Econometrics. 2021;225(2):200-30.

4.	Sun L, Abraham S. Estimating dynamic treatment effects in event studies with heterogeneous treatment effects. Journal of Econometrics. 2021;225(2):175-99.

5.	Cengiz D, Dube A, Lindner A, Zipperer B. The Effect of Minimum Wages on Low-Wage Jobs. The Quarterly Journal of Economics. 2019;134(3):1405-54.

6.	Li P, Lu Y, Wang J. Does flattening government improve economic performance? Evidence from China. Journal of Development Economics. 2016;123:18-37.

7.	Bo S, Wu Y, Zhong L. Flattening of government hierarchies and misuse of public funds: Evidence from audit programs in China. Journal of Economic Behavior & Organization. 2020;179:141-51.

8.	Yang X, Shi H-L. Specialization and product diversity. American Economic Review. 1992;82(2):392.

9.	La Porta R, Lopez‐de‐Silanes F, Shleifer A, w. vishny r. Law and Finance. Journal of Political Economy. 1998.

10.	Holmstrom B. Agency costs and innovation. Journal of Economic Behavior & Organization. 1989;12(3):305-27.

11.	Williamson OE. The New Institutional Economics: Taking Stock, Looking Ahead. Journal of Economic Literature. 2000;38(3):595-613.

12.	Dyck A, Zingales L. Private Benefits of Control: An International Comparison: Journal of Finance (Wiley-Blackwell). 2004;59(2):537-600.

13.	Edgerton J. Investment incentives and corporate tax asymmetries. Journal of Public Economics. 2010;94(11):936-52.

14.	Liu Q, Lu Y. Firm investment and exporting: Evidence from China's value-added tax reform. Journal of International Economics. 2015;97(2):392-403.

15.	Aghion P, Cai J, Dewatripont M, Du L, Harrison A, Legros P. Industrial Policy and Competition. American Economic Journal: Macroeconomics. 2015;7(4):1-32.

16.	Brandt L, Van Biesebroeck J, Zhang Y. Creative accounting or creative destruction? Firm-level productivity growth in Chinese manufacturing. Journal of Development Economics. 2012;97(2):339-51.

17.	Levine O, Warusawitharana M. Finance and productivity growth: Firm-level evidence. Journal of Monetary Economics. 2021;117:91-107.

18.	Liu X, Zeng J, Zhou Q. The chosen fortunate in the urbanization process in China? Evidence from a geographic regression discontinuity study. Review of Development Economics. 2019;23(4):1768-87.

19.	Melitz MJ, Polanec S. Dynamic Olley-Pakes productivity decomposition with entry and exit. The RAND Journal of Economics. 2015;46(2):362-75.

1. Referring to the method of Melitz and Polance [19], we define an entering firm as one that exists in the current year and does not exist in the previous year, and an exiting firm as one that exists in the current year and does not exist in the following year.
