# Supplementary material for: The impact of city-county merger on firm-level total factor productivity—Evidence from China
Source: PLoS One. 2024 Sep 16;19(9):e0304129. doi: 10.1371/journal.pone.0304129 (PMC11404814; doi:10.1371/journal.pone.0304129)
Supplement: Attachment — Submitted filename: Response to Reviewers-2024.4.28.docx [file pone.0304129.s002.docx]

**The Impact of City-county Merger on Firm-level Total Factor Productivity**

**Response to review #2**

We sincerely appreciate your engagement and time in reviewing our manuscript as well as your suggestions on improving it. Below there is a detailed account of how we have revised the manuscript based on each of your suggestions.

**Comment 1:** *Authors' have substantially revised the manuscript in light of the comments. However,* *the last paragraph of the paper seems to far fetched and the implications should be linked to the results. For example consider the following:
"In such scenarios, to bolster productivity and foster sustained economic growth, it is recommended that developing countries in the midst of economic transitions focus on enhancing the trade environment, improving the efficiency of intra-territorial transactions, and reducing firms' transaction costs. Moreover, providing government incentives to firms are also crucial."
How is the above flowing from the analysis?
Authors should revise the conclusion section and make it coherent.*

**Response to comment 1:** We appreciate your valuable suggestions. The baseline regression results indicate that TCD reform results in a significant decrease in the TFP of existing firms. Further analysis reveals that this finding is driven by the following mechanisms: (1) TCD reforms lead to a reduction in the institutional transaction efficiency, leading to a decline in firm-level TFP; (2) Following the reform, the local government may reduce tax incentives and other supportive policies for businesses, indicating that TCD reforms have worsened the external business environment, ultimately impacting firm-level TFP.

Based on the findings above, we have provided recommendations in the final paragraph of the paper to enhance transaction efficiency and increase government support for firms. To better align the recommendations with the findings, we have revised further the last paragraph. The detailed modifications to the last paragraph (page 30 of the revised manuscript) are as follows.

In addition, the insights gained from the experiences of the world’s largest developing country have significant implications for other nations with emerging markets. We have observed that TCD reforms result in a reduction in the efficiency of institutional transactions, leading to a decrease in firm-level total factor productivity (TFP). Therefore, we suggest that developing countries undergoing economic transitions prioritize efforts to improve the effectiveness of intra-territorial transactions by expanding the provision of public services such as infrastructure. Moreover, our findings demonstrate that changes in the external policy environment can impact the internal operating environment of firms. In conclusion, policymakers should strive to maintain policy coherence and reduce policy uncertainty to mitigate the impact of policy changes on firm operations.
